# Health Tract, No. 113

**Published:** 1862-11

**Authors:** 


### THE MORNING-PRAYER.

The humble and consistent looking upward for the gratification of our desires, the satisfaction of our wants, and that aid which comes from above to enable us to perform properly all the duties of life, is a religious obligation. But Providence has so arranged matters, that the performance of our duties may bring great benefits along with it. Many of the "observances" which Moses imposed upon the Israelites tended directly to the promotion of human health, of physical well-being. Moldy, spotted houses, damp and disease-engendering, were to be pulled down, and their materials scattered or burned; frequent personal ablutions were insisted on, thereby promoting individual healthfulness; while the use of rank meats, and other articles of food, unsuited to that climate, was most specifically prohibited. The disuse of all flesh for a month or more in the spring of the year, in some religious denominations, is the dictate of a sound physiology, and is not only promotive of health, but is antagonistic of disease; and if it were wisely carried out for "forty days" every spring, would demonstrably prevent many an attack of sickness, and would extend many a valuable life. Numerous spring diseases are directly traceable to the undisputed physiological fact that, as the warm weather approaches, we need one third less food, and sickness is inevitable when as much is eaten in warm weather as in cold. A judiciously observed "fast" is as promotive of physical as of spiritual health. There is wisdom and piety in the early morning-prayers of some churches; and there is health in them too! A multitude of moral, social, and physical good effects would follow, if in all large towns and cities fifteen minutes were spent in singing and prayer in every house of worship, at some convenient early hour. Ten verses might be read, three or four stanzas of some familiar hymn sung, and a short, pertinent prayer offered by the clergyman, some of his officers, or other active Christian men, to commence at the moment and end with the fifteen minutes by the stroke of a bell. The merchant, on his way down to his store; the lawyer, to his office; the workman, to his shop; the banker, to his desk—all could easily arrange to stop in and carry on with them a sanctifying influence to impregnate all the after-business transactions of the day. The son or daughter, on their way to school, could accompany their father; and a walk, on such a mission, to the mother or grown daughter and son, soon after breakfast, how it would break up the "second naps" of the morning, and that lazy, late lounging in bed, which saps the health and vitiates the habits of so many of the young of cities! Such a plan would waken up early activities by presenting an object for the same; would infuse a new life into our morning existence, and give many an hour of out-door exercise to our wives and daughters, for want of which many of them prematurely pine away and die. Such meetings would create a neighborly feeling among the members of many congregations; would promote unity and love and coöperation in building up the interests of the Church; would bring the members nearer together, and would be a bond of social and Christian union of incalculable value, besides the hygienic advantages already stated.

The ready plea of want of time is not valid. There is not a man in New-York who could not save fifteen minutes from any day's work and give it to the morning prayer-meeting. As for our wives and grown daughters, many of them are literally dying off in-doors, for want of an adequate inducement to dress and go out in the open air, pleasantly, for an hour or two a day. Such an expenditure of time daily, systematically, would add years to the life of some, and save others from weary weeks and months of worse than idleness on beds of avoidable sickness, because they not only lose their own time, but require that of others to attend them, besides deranging the movements of the whole household.

